# Effects of Simulated Marker Placement Deviations on Running Kinematics and Evaluation of a Morphometric-Based Placement Feedback Method

**DOI:** 10.1371/journal.pone.0147111

**Published:** 2016-01-14

**Authors:** Sean T. Osis, Blayne A. Hettinga, Shari Macdonald, Reed Ferber

**Affiliations:** 1 Faculty of Kinesiology, University of Calgary, Calgary, Canada; 2 Faculty of Nursing, University of Calgary, Calgary, Canada; 3 Running Injury Clinic, Calgary, Canada; Universidad Europea de Madrid, SPAIN

## Abstract

In order to provide effective test-retest and pooling of information from clinical gait analyses, it is critical to ensure that the data produced are as reliable as possible. Furthermore, it has been shown that anatomical marker placement is the largest source of inter-examiner variance in gait analyses. However, the effects of specific, known deviations in marker placement on calculated kinematic variables are unclear, and there is currently no mechanism to provide location-based feedback regarding placement consistency. The current study addresses these disparities by: applying a simulation of marker placement deviations to a large (*n = 411*) database of runners; evaluating a recently published method of morphometric-based deviation detection; and pilot-testing a system of location-based feedback for marker placements. Anatomical markers from a standing neutral trial were moved virtually by up to 30 mm to simulate deviations. Kinematic variables during running were then calculated using the original, and altered static trials. Results indicate that transverse plane angles at the knee and ankle are most sensitive to deviations in marker placement (7.59 degrees of change for every 10 mm of marker error), followed by frontal plane knee angles (5.17 degrees for every 10 mm). Evaluation of the deviation detection method demonstrated accuracies of up to 82% in classifying placements as deviant. Finally, pilot testing of a new methodology for providing location-based feedback demonstrated reductions of up to 80% in the deviation of outcome kinematics.

## Introduction

Anatomical marker placement has been identified as the single largest source of variance in repeated gait analyses using motion-capture techniques, resulting in inter-examiner differences of up to 34 degrees for certain joint angles [1]. This problem is particularly relevant for large, multi-site biomechanical gait studies where inter-tester differences can preclude pooling of data [2, 3]. To address this problem, a novel method was developed which ‘scores’ marker placement by comparing marker data transformed by generalized-Procrustes analysis (GPA), to a large normative database [4].

The outcome from this analysis is known as an inter-quartile range ratio (IQRR), and is a standardized, non-parametric, statistical description of the location of a given marker placement. It has been shown that the IQRR is capable of indicating divergence in marker placements between a single Novice and Expert, and measurements over time can show how a single Novice alters their placements [4]. While these results provide valuable insights, there is a lack of contextual information about how downstream kinematic data may be affected by marker deviations described by the IQRR. Therefore, two questions arise: 1) which anatomical markers have the greatest influence over outcome kinematics, and 2) what is an ‘acceptable’ value for the IQRR at each location to indicate that deviations in marker placement are within some acceptable limit.

A simulation approach has produced valuable descriptions of deviation effects for knee markers [5], and propagation into coordinate systems [6]. However, these studies did not comprehensively address the impact of specific marker placement deviations on lower extremity kinematics of gait analysis, particularly in light of anatomical variance. By simulating deviations in marker placement, at multiple locations, for a large subject cohort, it is possible to determine the worst-case effects of these deviations. Additionally, by considering the IQRR score as a binary classifier of deviation, conventions can be established for what constitutes ‘acceptable’ or ‘unacceptable’ IQRR scores.

Therefore, the purposes of this study were to:

1. Use simulated deviations in marker placement to calculate resulting changes in downstream gait kinematics and quantify the relationships between marker placement deviations and changes in kinematic variables
2. Evaluate the classification performance of IQRR scores by considering kinematic changes in terms of Mathews’ correlation coefficient (MCC), false positive rates (fall-out), and true positive rates (sensitivity) for a range of kinematic and IQRR thresholds
3. Conduct a real-world pilot study of IQRR-based deviation detection with novice biomechanists.

## Materials and Methods

### Data Collection

A total of *n = 411* subjects were included in this study (gender: *215 females*; age: *40*.*0 ± 11*.*6 yrs*; height: *172*.*3 ± 8*.*9 cm*; mass: *71*.*2 ± 13*.*1 kg*). All were patients who participated in clinical activities at the Running Injury Clinic, and all gave written, informed consent for their data to be used for research purposes. Patients presented with a variety of common running injuries (i.e. patellofemoral pain syndrome, iliotibial band syndrome), however, none presented with conditions known to drastically influence lower body morphology (i.e. amputation, developmental disorders, lower extremity orthopaedic surgery, etc). Inclusion criteria were kept intentionally broad in order to represent a large segment of the population in a morphological sense, thereby improving the generalizability of the method. Ethical approval was given by the Conjoint Health Research Ethics Board to review and analyze patient data for the current study.

Three-dimensional (3D) kinematics were collected on each patient using 9.5 mm spherical retro-reflective markers and eight high-speed digital video cameras recording at 200 Hz (MX-3, Vicon, Oxford, UK). Cameras were placed in a uniformly-spaced circular arrangement, surrounding the treadmill. Markers were placed over anatomical landmarks to create an anatomical model of the lower extremities according to previously published methods [7, 8]. Subjects wore tight-fitting shirts and shorts, so as to limit the impact of clothing on marker placement. All marker placements were performed by one tester (the Expert) with 15 years of experience in clinical anatomy and more than 800 3D kinematic gait analyses performed.

In brief, the marker model used for the lower extremity consisted of the following conventions. Ankle joint centres were located at the midpoint of the lateral and medial malleoli markers. Knee joint centres were located at the midpoint of markers placed over the lateral and medial joint lines. Hip joint centers were determined using trochanter markers and locating them at 25% of the inter-trochanteric distance [9].

A joint coordinate system [10] was used to construct segment-aligned coordinate systems, relative to segment tracking markers. Briefly, each segment coordinate system was created by aligning the long axis of the segment with the vector connecting adjacent joint centres. The anterior axis was then calculated from the cross product of the long axis and the vector connecting medial and lateral markers of the distal joint. Finally, the hinge axis was calculated as the cross-product of the anterior and long axes. Segment tracking clusters were affixed to the pelvis, thighs, shanks and feet.

Each subject performed a standing neutral trial with all anatomical markers to establish the anatomical model, and their position was standardized using a template under their feet. Once the standing trial data were collected, the anatomical markers were removed, however the segment tracking markers were left on the subject for the dynamic trial. The treadmill was then sped up to a comfortable jogging speed between 5.5 and 6.5 mph. The subject was given a 2–5 minute period of acclimatization, after which a 20–60 second dynamic running trial was collected. All data were post-processed in MATLAB (The Mathworks, Natick, USA) using custom software according to the following procedures.

### Error Sensitivity

For each subject, a set of 29 standing trials was created from their original standing trial. One of the 29 trials consisted of the unaltered trial, and the remaining 28 trials each had a randomly simulated deviation added to either the vertical or anteroposterior coordinate for ONE of the following markers: greater trochanters (bilaterally), medial and lateral knee markers (bilaterally), medial and lateral ankle markers (bilaterally) and medial and lateral metatarsophalangeal markers (bilaterally), for a total of 28 marker-coordinates. Errors (*e*) were randomly drawn from the uniform distribution over the interval -30 mm < *e* < 30 mm, as suggested by Stagni et al. [11].

Marker data from the running trials were low-pass filtered using a recursive Butterworth filter with a 10 Hz cutoff. Kinematics for segment tracking clusters were calculated at the ankle, knee and hip, from the standing and running trials using a singular-value decomposition method [12] and a joint coordinate system [10]. Angular velocities were calculated by differentiating joint angles. A selection of discrete variables was then calculated from the kinematic data, which consisted of typical peaks and excursions reported in clinical biomechanics literature [13, 14, 15, 16]. This procedure was repeated with each of the 29 standing trials, using the same running trial each time. Kinematic change was defined as the absolute difference between outcome kinematics for the unaltered standing trial and the outcome kinematics from each of the 28 standing trials with error introduced.

In order to compare relationships between marker placement errors and downstream changes in kinematics, each kinematic change was standardized to 10 mm (approximately one marker width) of placement error. These kinematic change ratios were therefore calculated in units of degrees/10 mm of error for angles, or degrees per second/10 mm of error for angular velocities. This transformation of the data resulted in *n = 411* normally distributed ratios for each marker/variable pair. A point estimate of the 95%ile of the ratio was calculated for each marker/variable pair using a weighted average of the closest two values to the 95% percentile.

The entire simulation was repeated 10 times using the same procedure, but drawing different random errors from the uniform distribution. The 95%ile ratios calculated for marker/variable pairs for each of the 10 iterations were averaged to produce a mean point-estimate of the 95%ile change ratio.

### IQRR Classifier Evaluation

A leave-one-out cross validation approach was used to assess IQRR classifier performance. Each of the 29 static trials used in the simulation were also spatially normalized using the modified GPA procedure [4] using *n = 411–1* standing trials as the reference database, leaving-out the standing trial being analyzed. Briefly, a subset of reference data was selected from the reference database using a majority-vote, nearest-neighbour analysis, and the IQRR for each coordinate-marker pair was calculated according to previously published methods [4].

Using the results of the error simulation, kinematic change in selected discrete variables was compared with the IQRR scores from the GPA. Confusion matrices were constructed by dichotomizing kinematic variable data and IQRR scores using thresholds, which were iteratively set. For IQRR scores, thresholds were set at increments of 0.1 from 0.0 to 1.0. For kinematic change, thresholds were set at 0.1 degree increments from 0 to 15 degrees (beyond which less than 0.5% of cases occurred). At each pair of thresholds, a corresponding confusion matrix was generated by calculating true-positives, false-positives, true-negatives and false-negatives, with respect to the thresholds set. If any category of the confusion matrix consisted of less than five instances for a given threshold, this was identified as a marginal case and this threshold pair was discarded. This procedure was repeated a total of 10 times using the marker errors from each iteration of the error simulation to produce a total of 10 confusion matrices for each threshold pair.

For each confusion matrix, the Matthew’s correlation coefficient (MCC) was calculated as a single descriptor of classifier performance for a given pair of kinematic change and IQRR score thresholds. A point estimate of the mean MCC for a threshold pair was calculated as the average of MCCs across the 10 iterations of the error simulation. This generated a 10 x 150 matrix of mean MCCs corresponding to each combination of thresholds. The maximum mean MCC was found in order to identify the pair of thresholds that produced the most balanced classifier performance.

### Real-World Pilot Study

In order to support a real-world application of the IQRR scoring method and thresholds, a pilot test was conducted using one test subject with several marker placements. One placement was performed by the aforementioned Expert and six more marker placements were conducted by Novices with anatomical knowledge, but no prior biomechanics experience. Each Novice was given a schematic indicating the placements, and the anatomical locations were described verbally. Segment tracking clusters were placed only once, therefore being identically located for all trials.

**Fig 1:**
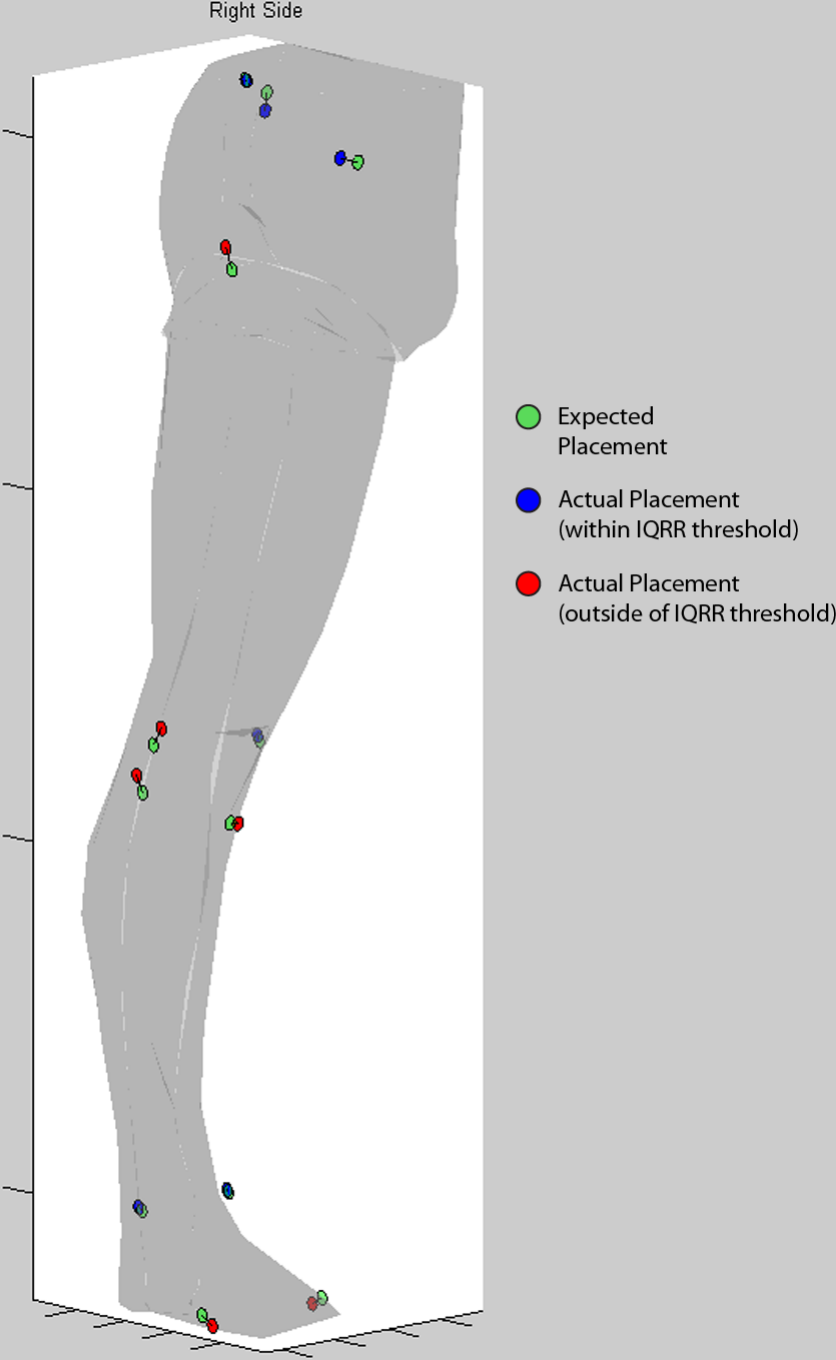
Feedback tool screenshot. Screenshot demonstrating feedback given to the Novices of the pilot study to indicate potential errors in marker placement. A lower-limb surface model was constructed and anatomical marker position data was overlaid to provide context for users (segment markers locations were identical for all trials, and therefore omitted). The marker position data consisted of expected positions (green circles), which were scaled to fit the surface model, and dimensionless IQRR scores (blue and red circles), which were scaled and positioned relative to the expected position with a connecting line to indicate directionality. Red circles indicated that a marker had crossed the threshold of 0.8 for the associated IQRR score, while blue circles indicated the marker was within the threshold. Participants were instructed to only modify those placements indicated by red circles, and to use their judgment in deciding how much to move the marker.

Within 30 seconds of the first placement and standing trial by each Novice, standardized feedback was given regarding their placement. A 3D plot was presented, showing the expected location of anatomical markers, and the location of the Novice placements in “IQRR space” for the anteroposterior and vertical coordinates (Fig 1). The data were presented in the context of a 3D surface model of the lower limb to provide visual guidance, however, no scale or measurement information was given. A threshold of 0.8 was chosen as a cutoff for the IQRR score based on the findings from the IQRR classifier evaluation. Novices were instructed to only change markers for which the IQRR of 0.8 was exceeded, and to use their judgment in making modifications. After placements were modified, a second standing trial was taken. After all marker placements by the Expert and Novices were completed, the test subject performed a running trial, which was then analyzed using all 13 standing trials.

The kinematic change or deviation from the Expert data was calculated as the difference between the discrete kinematic variables of the Expert from those calculated based on Novice marker placements. Median deviation was then determined both prior to feedback and afterwards, across both left and right sides for 9 kinematic variables: peak ankle abduction, internal rotation, dorsiflexion, peak knee abduction, external rotation, flexion, and peak hip adduction, internal rotation and extension. Wilcoxon Signed-Rank tests were performed on all 9 variables and tests were adjusted using the Bonferroni-Holmes procedure, with a family-wise alpha of 0.05.

## Results

### Error Sensitivity

**Fig 2:**
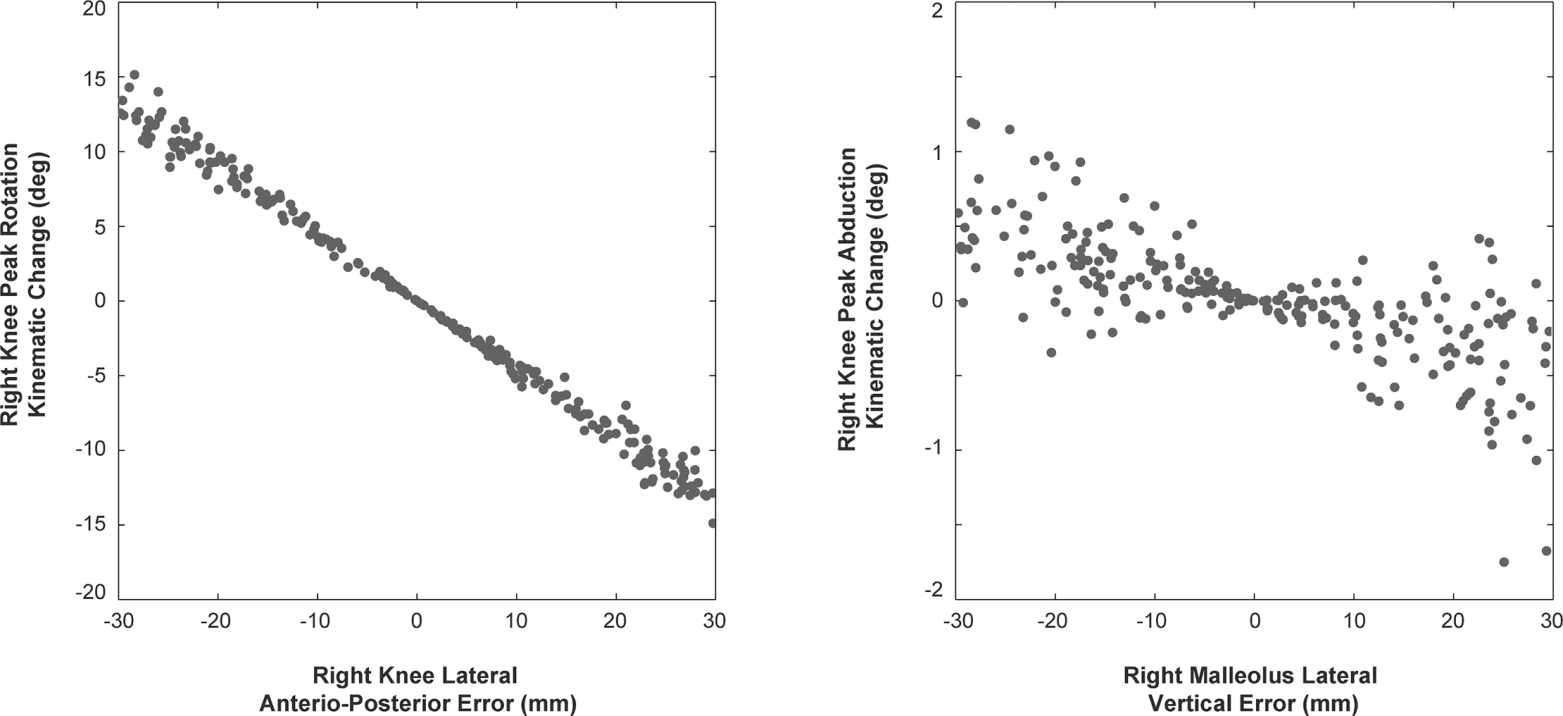
Relationships between marker errors and changes in discrete variables. Two basic relationships were observed: the left panel shows a highly linear relationship between anteroposterior marker placement error and kinematic change in the transverse plane, the right panel shows a non-linear relationship between vertical marker error and frontal plane kinematics.

In general, errors in the anteroposterior direction exhibited clear linear relationships with variables in the transverse plane, along with much larger 95%ile change ratios than other planes (Fig 2). Conversely, errors in the vertical coordinate tended to exhibit less clearly defined relationships with frontal plane variables.

**Fig 3:**
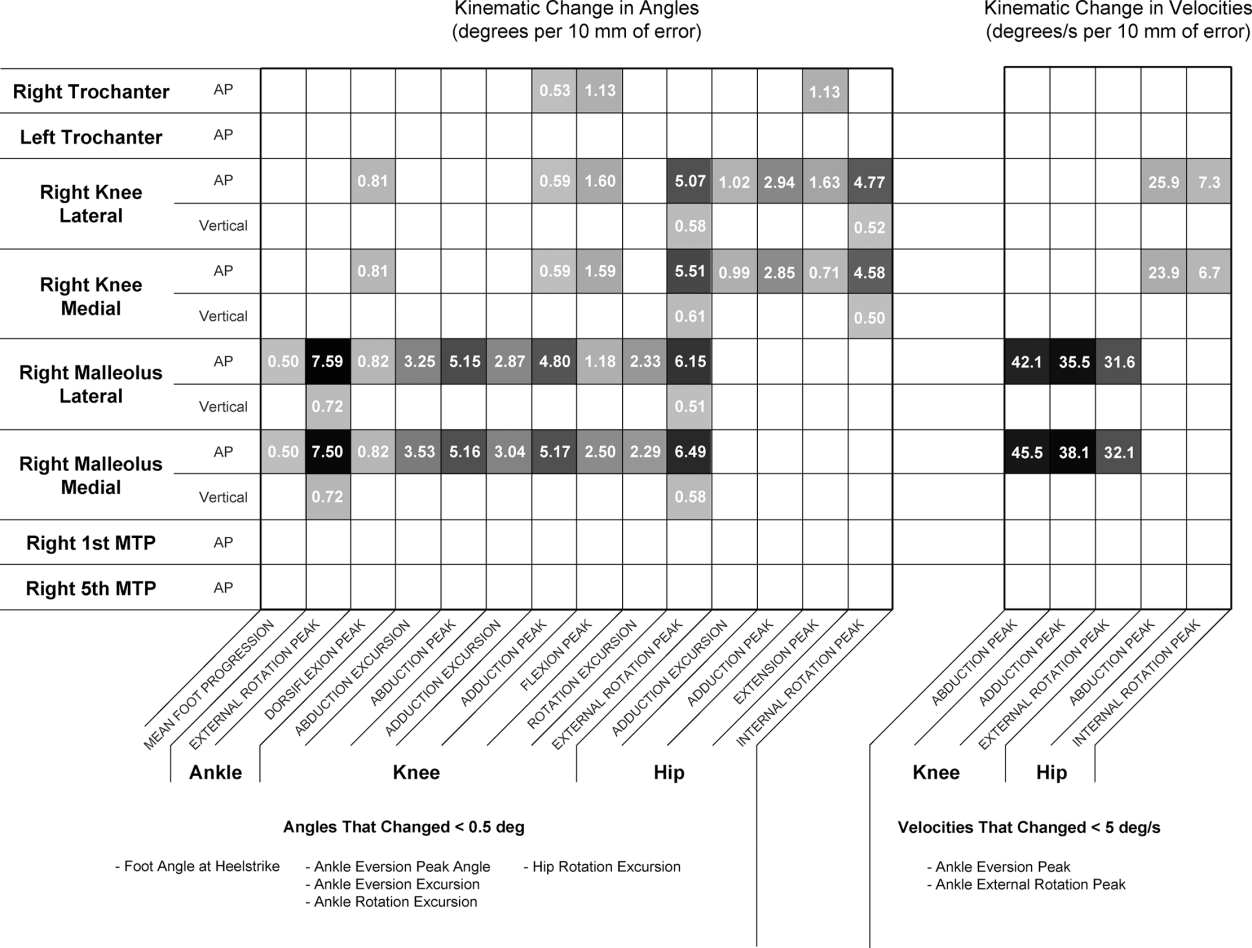
Tabulated results of the error sensitivity analysis. Values shown are for right side kinematics only, however, left side trends were nearly identical. All values for kinematic change ratios represent a point estimate of the 95th percentile ratio, using the mean of 10 iterations of the simulation. All values shown are in normalized units of degrees of error per 10 mm of marker error (for angles), or degrees/s of error per 10 mm of marker error (for angular velocities). Empty cells indicate that a variable changed less than 0.5 degrees, or 5 degrees/second, for every 10 mm of error.

Knee and ankle markers demonstrated influence over the largest number of kinematic variables, with trochanter markers and MTP markers influencing only a few variables (Fig 3). In terms of peak angles, the anteroposterior (AP) coordinates of the knee and ankle markers produced the largest angular changes (up to 7.59 degrees/10 mm). In terms of angular velocities, errors in AP ankle markers produced the largest changes (up to 45.5 degrees per second/10 mm).

### IQRR Classifier Evaluation

There was no single IQRR threshold that produced maximum MCCs across all variable/marker pairs, therefore maximum MCCs were identified for selected IQRR thresholds to evaluate potential performance benefits (Tables 1–3). An IQRR threshold of 0.8 maximized true positive rates (sensitivity) across nearly all of the marker-coordinate pairs, whereas larger tradeoffs were apparent for other threshold criteria.

**Table 1:** Tabulated results of the evaluation of the IQRR as a classifier of placement deviation, using maximum MCC.

*Marker Coordinate*	*Discrete Variable*	*Threshold for IQRR Score*	*Threshold for Kinematic Change*	*Maximum MCC*	*Accuracy*	*True Positive Rate (Sensitivity)*	*False Positive Rate (Fall-Out)*
*Right Trochanter AP*							
	*Hip Extension Peak Angle*	*0*.*9*	*1*.*8 deg*	*0*.*3410*	*0*.*6800*	*0*.*6269*	*0*.*2837*
	*Knee Flexion Peak Angle*	*0*.*9*	*1*.*7 deg*	*0*.*3405*	*0*.*6776*	*0*.*6183*	*0*.*2782*
*Right Knee Lateral AP*							
	*Hip Adduction Peak Angle*	*0*.*9*	*2*.*3 deg*	*0*.*4345*	*0*.*7231*	*0*.*7428*	*0*.*3052*
	*Hip Internal Rotation Peak Angle*	*0*.*8*	*6*.*3 deg*	*0*.*4895*	*0*.*7443*	*0*.*7634*	*0*.*2736*
	*Knee External Rotation Peak Angle*	*0*.*9*	*7*.*0 deg*	*0*.*4850*	*0*.*7416*	*0*.*7669*	*0*.*2812*
*Right Malleolus Lateral AP*							
	*Ankle External Rotation Peak Angle*	*1*.*0*	*6*.*8 deg*	*0*.*5837*	*0*.*8185*	*0*.*9138*	*0*.*3651*
	*Knee Abduction Peak Angle*	*0*.*9*	*3*.*7 deg*	*0*.*5224*	*0*.*7844*	*0*.*9039*	*0*.*4209*
	*Knee Adduction Peak Angle*	*1*.*0*	*1*.*7 deg*	*0*.*5231*	*0*.*8131*	*0*.*8649*	*0*.*3338*

**Table 2:** Tabulated results of the evaluation of the IQRR as a classifier of placement deviation, using an IQRR threshold of 0.9 and finding maximum MCC.

*Marker Coordinate*	*Discrete Variable*	*Threshold for IQRR Score*	*Threshold for Kinematic Change*	*Maximum MCC*	*Accuracy*	*True Positive Rate (Sensitivity)*	*False Positive Rate (Fall-Out)*
*Right Trochanter AP*							
	*Hip Extension Peak Angle*	*0*.*9*	*1*.*8 deg*	*0*.*3396*	*0*.*6854*	*0*.*5710*	*0*.*2365*
	*Knee Flexion Peak Angle*	*0*.*9*	*1*.*7 deg*	*0*.*3383*	*0*.*6805*	*0*.*5623*	*0*.*2315*
*Right Knee Lateral AP*							
	*Hip Adduction Peak Angle*	*0*.*9*	*2*.*3 deg*	*0*.*4345*	*0*.*7231*	*0*.*7428*	*0*.*3052*
	*Hip Internal Rotation Peak Angle*	*0*.*9*	*5*.*7 deg*	*0*.*4846*	*0*.*7440*	*0*.*7854*	*0*.*3033*
	*Knee External Rotation Peak Angle*	*0*.*9*	*5*.*6 deg*	*0*.*4849*	*0*.*7472*	*0*.*7660*	*0*.*2787*
*Right Malleolus Lateral AP*							
	*Ankle External Rotation Peak Angle*	*0*.*9*	*6*.*5 deg*	*0*.*5697*	*0*.*8173*	*0*.*9253*	*0*.*4032*
	*Knee Abduction Peak Angle*	*0*.*9*	*3*.*4 deg*	*0*.*5075*	*0*.*7900*	*0*.*9084*	*0*.*4457*
	*Knee Adduction Peak Angle*	*0*.*9*	*1*.*7 deg*	*0*.*5229*	*0*.*8192*	*0*.*8871*	*0*.*3741*

**Table 3:** Tabulated results of the evaluation of the IQRR as a classifier of placement deviation, using an IQRR threshold of 0.8 and finding maximum MCC.

*Marker Coordinate*	*Discrete Variable*	*Threshold for IQRR Score*	*Threshold for Kinematic Change*	*Maximum MCC*	*Accuracy*	*True Positive Rate (Sensitivity)*	*False Positive Rate (Fall-Out)*
*Right Trochanter AP*							
	*Hip Extension Peak Angle*	*0*.*8*	*1*.*8 deg*	*0*.*3410*	*0*.*6800*	*0*.*6269*	*0*.*2837*
	*Knee Flexion Peak Angle*	*0*.*8*	*1*.*7 deg*	*0*.*3405*	*0*.*6776*	*0*.*6183*	*0*.*2782*
*Right Knee Lateral AP*							
	*Hip Adduction Peak Angle*	*0*.*8*	*1*.*9 deg*	*0*.*4310*	*0*.*7338*	*0*.*7600*	*0*.*3171*
	*Hip Internal Rotation Peak Angle*	*0*.*8*	*5*.*6 deg*	*0*.*4806*	*0*.*7428*	*0*.*8236*	*0*.*3528*
	*Knee External Rotation Peak Angle*	*0*.*8*	*5*.*1 deg*	*0*.*4786*	*0*.*7526*	*0*.*7912*	*0*.*3102*
*Right Malleolus Lateral AP*							
	*Ankle External Rotation Peak Angle*	*0*.*8*	*6*.*5 deg*	*0*.*5573*	*0*.*8129*	*0*.*9435*	*0*.*4537*
	*Knee Abduction Peak Angle*	*0*.*8*	*3*.*4 deg*	*0*.*4959*	*0*.*7866*	*0*.*9275*	*0*.*4936*
	*Knee Adduction Peak Angle*	*0*.*8*	*1*.*7 deg*	*0*.*5068*	*0*.*8192*	*0*.*9066*	*0*.*4299*

Each marker/variable pair was evaluated on IQRR thresholds from 0.0 to 1.0 (0.1 increments) and on kinematic change thresholds from 0.0 to the maximum value (0.1 degree increments). Table 1 includes results for the maximum MCC scores found across all threshold pairings. Table 2 includes results when the IQRR threshold was fixed at 0.9 and maximum MCC was found for kinematic change. Table 3 includes results when the IQRR was fixed at 0.8 and maximum MCC was found for kinematic change.

### Real-World Pilot Study

**Fig 4:**
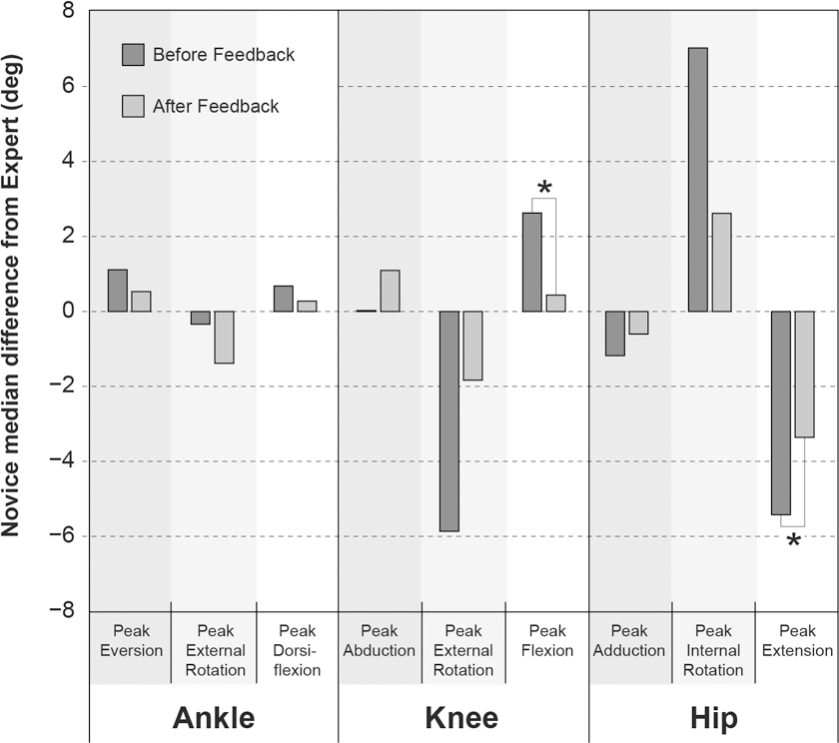
Results of the pilot study. Bars indicate the signed median difference between the group of n = 6 Novices and the Expert, both before and after receiving feedback. * indicates significant differences from the Signed Wilcoxon Rank test (family-wise p <0.05).

Median deviation in kinematic variables between Novice testers and the Expert trended towards zero in 7 of the 9 kinematic variables after feedback was given (Fig 4). However, these differences were only significant for peak knee flexion and peak hip extension (family-wise α = 0.05).

## Discussion

The purposes of this study were to: 1) use simulated deviations in marker placement to calculate resulting changes in downstream kinematic variables and quantify relationships between marker placement and changes in kinematic variables; 2) evaluate the classification performance of the IQRR score by considering MCCs, false positive (fall-out) and true positive (sensitivity) rates for a range of kinematic and IQRR thresholds; and 3) to conduct a real-world pilot study of the IQRR method. The results indicate large influences of malleolus and knee marker placement deviations on kinematics, along with favorable performance of the IQRR classifier in detecting markers placed in different (unexpected) locations.

It has previously been demonstrated that deviation in marker placement can affect many downstream variables [5], and that precision in the 3D coordinates of anatomical landmarks propagates into joint angle calculations for the lower limb [6, 17]; however, specific relationships between the two have not been elucidated. According to current results, it is not only knee markers that can have a large influence over kinematic variables, but ankle marker placement as well. In both cases, the most affected joint angles were those in the transverse plane at the hip, knee and ankle, while the most affected velocities were in the frontal and transverse planes at the knee joint. These findings are consistent with experimental studies that have also highlighted the sensitivity of variables in the transverse plane [2, 18, 19]. Taken together, these results argue for caution in measuring kinematics of the transverse plane.

Previous studies have postulated that relationships between marker placement and gait kinematics arise from conventions in the creation of segment coordinate systems [1, 6, 17, 20]. Indeed, it is likely that the conventions chosen will determine the propagation of deviations, and therefore, generalizations from the current findings must be interpreted in the context of the model used. For many segment-fixed joint coordinate systems (JCS), the joint centre and the hinge axis are defined using two markers on the lateral and medial sides of a joint, making these JCS hinges highly sensitive to anteroposterior deviations.

Since joint angles are calculated based upon orientations of the two segments they connect, the construction of either segment coordinate system may exert an influence over the calculated joint angle. It is therefore understandable that malleoli markers influence ankle and knee transverse angles, but not hip angles, while knee markers primarily influence hip and knee transverse angles (Fig 3). Knee, ankle and hip markers also influence sagittal plane joint angles, and this effect likely arises from the construction of the long axes of segments based on the location of the proximal joint centres. Although this effect is much smaller than effects in other planes, it is worth noting that prior studies have demonstrated small but significant differences in sagittal plane joint angles between testers [1], and it is possible that these differences can be entirely accounted for by this mechanism.

Prior studies of placement deviation have asserted that marker placement deviations are unpredictable [17], and indeed, it is not eminently clear how these deviations occur. To this point, by using a large cohort this study provides near-worst-case 95^th^ percentile changes in a given kinematic variable in response to a 10 mm placement deviation. The standard value of 10 mm was chosen as this approximates one marker diameter (9.5 mm markers were used in this study). This ‘rule-of-thumb’ can be applied in context to provide an estimate of whether a kinematic difference might be spurious. As a simple example, if a researcher or clinician is confident they rarely deviate in malleoli markers by more than half-a-marker-width, then they can infer that a repeated measures difference in peak ankle rotation which exceeds 3.80 degrees would fall outside of the 95%ile error estimate (7.59 degrees/10 mm error x 1/2). It is critically important to qualify, however, that the validity of this approach is dependent upon the lower extremity model chosen by the investigators.

As a binary classifier of placement errors, the IQRR score shows promise as a means of deviation detection. The fixed IQRR threshold of 0.8 results in very good sensitivity (true positive rates of ~62–94%), with reasonably low corresponding thresholds for many discrete kinematic variables (Table 3). The specificity of the IQRR score is not as high (true negative rates of ~51–72%), however this is not entirely unexpected. It is clear that the IQRR score does not reflect only deviations in placement, but rather a combination of deviation and unique anatomical configuration [4]. Given this feature of the analysis, and the high false positive (fall-out) rates, the results cannot be taken as an absolute measure of placement deviation, but rather as a valuable training and reference tool.

Pilot data using placement feedback demonstrated that a group of Novices with no biomechanics experience were able to immediately reduce their median deviation from the Expert by up to ~80% for knee sagittal kinematics. In two individual cases, there were consistent reductions, of up to 11 degrees, in deviations from Expert kinematics. This result, however, was not universal, and there appeared to be a tendency in two other Novices to over-adjust their placements. Further work is therefore needed to optimize feedback and to study longitudinal learning trends over many uses of the feedback tool. Future studies involving: a larger sample of novice users, multisite comparisons, and dynamic events other than running gait are also necessary.

A principal limitation of the current study is the reference database from which the feedback were generated. The nature of this reference database will define the limits of its applicability in terms of specific morphologies (i.e. developmental disorders, surgical changes, diseases affecting bone structure). However, while this method may currently be limited in application to a population of morphologically average individuals, the database may easily be scaled to include more individuals as data are collected on them. This is a significant strength of the current method, as the database will continue to evolve and expand as the method is applied in practice.

In conclusion, important and identifiable relationships exist between marker placement deviations and downstream kinematics, placement classification based on the IQRR score detected up to 94% of simulated deviations, and pilot testing of a placement feedback tool by Novices resulted in significant reductions in their deviation from results obtained by the Expert. Thus, the current study supports the use of placement deviation detection and feedback, and speaks to the need for future research in evaluating the utility and optimal application of this approach.

## Supporting Information

S1 Dataset Comprehensive results from iteration 1 of the simulation experiment. Each iteration of the simulation is saved as a separate file. Within each file, individual spreadsheets contain data from each subject. Within each spreadsheet, rows 3–30 contain calculated discrete variables from the unaltered trials and with errors added to each marker/coordinate, and rows 34–60 contain IQRR scores from the marker placement GPA calculations.
(XLSX)

S2 Dataset Comprehensive results from iteration 2 of the simulation experiment. (XLSX)

S3 Dataset Comprehensive results from iteration 3 of the simulation experiment. (XLSX)

S4 Dataset Comprehensive results from iteration 4 of the simulation experiment. (XLSX)

S5 Dataset Comprehensive results from iteration 5 of the simulation experiment. (XLSX)

S6 Dataset Comprehensive results from iteration 6 of the simulation experiment. (XLSX)

S7 Dataset Comprehensive results from iteration 7 of the simulation experiment. (XLSX)

S8 Dataset Comprehensive results from iteration 8 of the simulation experiment. (XLSX)

S9 Dataset Comprehensive results from iteration 9 of the simulation experiment. (XLSX)

S10 Dataset Comprehensive results from iteration 10 of the simulation experiment. (XLSX)
